# Unusual cup reconstruction of massive acetabulum perforation in neglected femoral neck osteoporotic fracture: A case report

**DOI:** 10.1016/j.ijscr.2019.06.019

**Published:** 2019-06-20

**Authors:** Franky Hartono, Karina Besinga, Daniel Petrus Marpaung, Andrew B. Budisantoso, Tessi Ananditya

**Affiliations:** a Pantai Indah Kapuk Hospital, Jakarta, Indonesia; b Siloam Kebon Jeruk Hospital, Jakarta, Indonesia

**Keywords:** Massive acetabulum perforation, Osteoporotic bone, Neglected femoral neck fracture, Case report

## Abstract

**Introduction:**

Uncemented total hip for neglected nonunion femoral neck fracture hopefully could minimize the post operative complications. Nevertheless, due to plasticity of the brittle osteoporotic bone, unexpected fracture may occur. We presented an unusual reconstruction of acetabulum which was accidently perforated during uncemented cup insertion.

**Presentation of case:**

A 76 years old wheelchair bound female showed a nonunion fracture of the right femoral neck due to a fall 7 years ago. She had right hip instability with 5  cm leg shortening. Uncemented total hip replacement was originally planned, however, due to unexpected perforation of the acetabulum, it was ended in total hip after creating a composite layers to fix a cemented cup. Patient was full ambulatory with walker at 4 months. Harris Hip Score at 15 months showed 71.3%, compared with 20.2% before surgery.

**Discussion:**

The plasticity of the osteoporotic acetabulum floor was the reason why the uncemented cup could not be fixed, instead it caused massive perforation of the floor. The acetabulum reconstruction was done by composite layers made from remaining chondral bone shell, absorbable hemostatic gelatin sponge and cancellous bone of the femoral head, porous collagen matrix and uncemented titanium acetabulum cup fixed with two screws.

**Conclusion:**

In unexpected acetabulum perforation, the remaining chondral shell of femoral head together with uncemented cup as a cage support and bone cement can be used to reconstruct the acetabulum floor. More stability can be achieved using reconstruction cage with allograft and or metal augments or using a cemented total hip arthroplasty.

## Highlights

• Neglected femoral neck fracture led to severe disuse osteoporos is resulting very brittle acetabulum floor and proximal femur shaft.
• The decision to use uncemented Total Hip Replacement was taken to reduce the risk of pulmonary embolism.
• Plasticity of the osteoporotic acetabulum floor caused bouncing effect in every single hit resulting in cup fixation failure and massive acetabulum perforation.
• In emergency situation, an acceptable result could be achieved by constructing an unusual cup composite, made from the remaining cancellous chondral shell of the femoral head as a base, the failed uncemented cup as a cage and a cemented cup fixed together with gentamycine loaded bone cement.

## 1 Introduction

Femoral neck fracture has a great impact on the blood supply of the femoral head especially if there are underlying disease as osteoporosis and geriatric metabolic and nutritional status.

Over two decades, the distribution of published reports of neglected femoral neck fracture has shifted to developing countries due to poverty, ignorance, lack of facilities or faith in traditional bonesetters [[1], [2], [3], [4]].

Uncemented acetabular components have been shown to have good success in osteoporotic individuals, especially in patients who had either high risk of infection or previous history of deep vein thrombosis or who had no cost constraints. This case report has been reported in line with the SCARE criteria [5].

## 2 Presentation of case

A 76 year-old woman was admitted in the emergency room with symptoms of weakness, nausea, stomachache, loss appetite and black stool since two days. The internist diagnosed her with stress ulcer. She was also experiencing pain in the right hip and unable to walk. Therefore, she was referred to our department. This woman had a medical history of falling on right hip 7 years ago that left untreated. Since that time she was wheelchair bound with chronic low back pain. No other significant medical history and no routine medication before she was hospitalized.

**Fig. 1:**
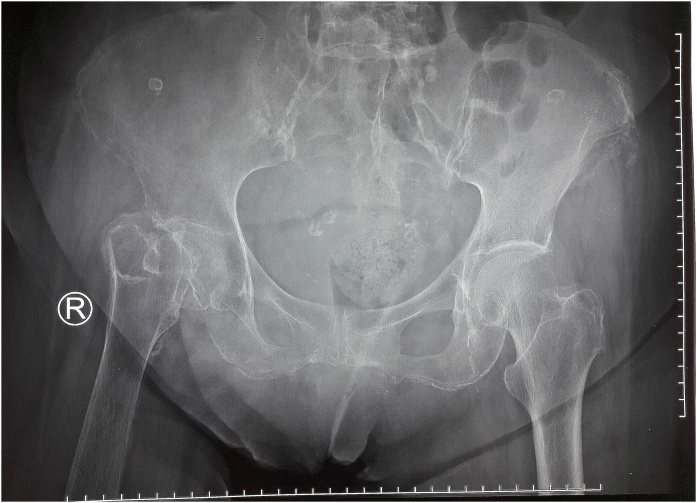
Preoperative showed Neglected Femoral Neck Fracture (Source: internal documentation).

**Fig. 2:**
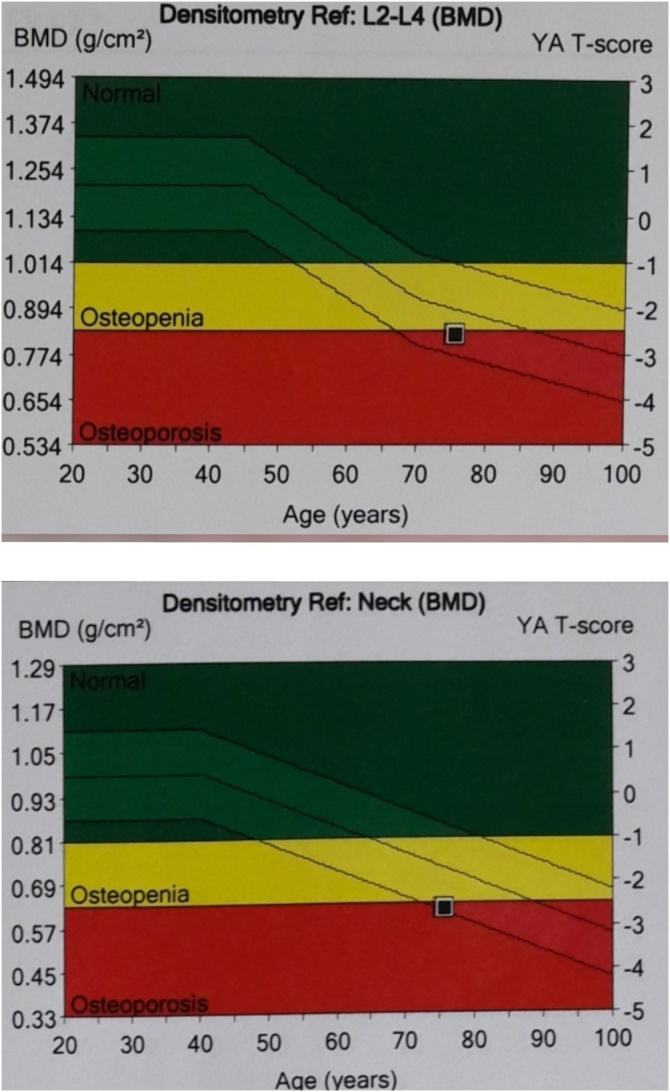
Patient’s Bone Mineral Densitometry showed osteoporotic bone (Source: internal documentation).

Telescoping test showed muscle weaknessalong with right hip instability and 5 cm leg length discrepancy. Pelvic X-ray revealed an disuse osteoporotic neglected nonunion fracture of the right femoral neck (Fig. 1) and BMD showed decreased bone mass density (Fig. 2). Lumbar X-ray showed a lumbar spondyloarthritis. Primary uncemented total hip arthroplasty with Smith and Nephew Reflection^®^ cup and Synergy^®^ femoral stem was planned. Preoperative template revealed the diameter 50 for acetabulum and 14 for femoral stem.

Under spinal anesthesia patient was positioned in lateral decubitus. The Kocher Langenbeck posterior approach was used to open the joint capsule. The remaining piece of the total eroded femoral head was removed. The femoral stem was reamed to prepare for implant size 14 without any problem. The acetabulum was reamed until size 48, which should fit for the cup size 50. Unfortunately, the acetabulum was perforated in the process of attaching an uncemented cup size 50 resulting a total floor defect of 5 cm diameter.

**Fig. 3:**
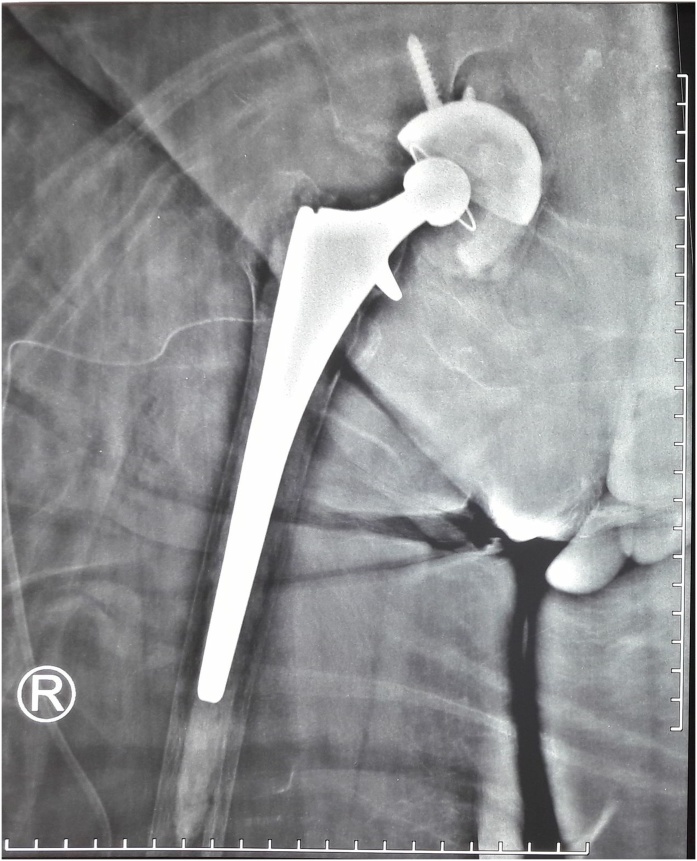
Post surgery result (source : internal documentation).

In the normal circumstance, our operating theater does not provide any reconstructive implants for acetabulum as a plate, cage or a wire mesh. To fix this unexpected problem, an alternative procedure was planned to build a solid base composite graft as a bed for cemented acetabulum cup. The composite was made from the remaining chondral shell of the femoral head and absorbable hemostatic gelatin sponge (Surgicell^®^) as the barrier wall between pelvic minor structure and the prosthesis, layered by mixture of remaining sparse cancellous bone of the femoral head, bone debris and porous collagen matrix (Osteovit^®^). The titanium uncemented acetabulum cup (Reflexion^®^), that previously planned as a final prosthesis, was fixed with two screws in that bed, acted now as a reconstruction cage. One pack of 40 mg gentamicin contained bone cement (Palacos^®^) was filled on top of that composite graft. A cemented poly-ethylene cup (Reflexion All-poly^®^) number 40–22 was fixed as the acetabulum prosthesis in the right inclination and anteversion position.. The cemented femoral stem size 14 was placed into the medullary of the femur without any problem (Fig. 3). The stability and length of the leg of this total hip replacement was assured by adjusting the appropriate length in the femoral neck prosthesis.

**Fig. 4:**
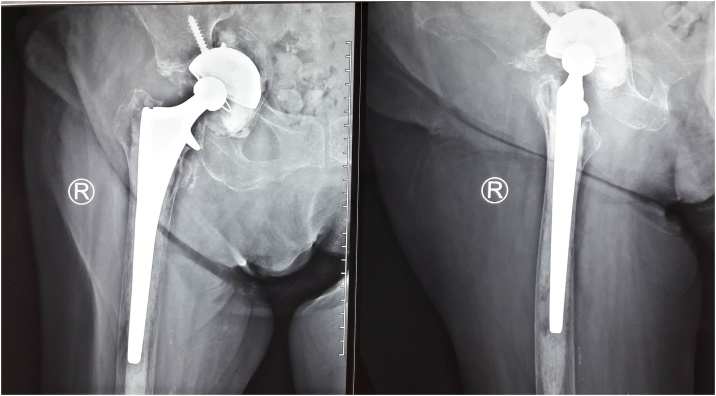
Three months post-operative showed density improvement (Source: internal documentation).

The patient had to rest in bed for six weeks after the surgery to consolidate the acetabulum composite. The X-Ray of the right hip at 3 months, showed the reformation of the acetabulum floor and stability of the prosthesis (Fig. 4).

## 3 Discussion

Neglected femoral neck fracture leads to severe disuse osteoporosis. Uncemented total hip replacement was preferred because it is simpler, has less operating time, in addition could diminish post operative complications such as deep vein thrombosis or pulmonary embolism and bone cement implantation syndrome, although it has potential complications as intraoperative fracture.

We did not encounter problem in the placement of cemented Synergy^®^ femoral prosthesis, but well during preparation of the acetabulum.

A massive acetabulum perforation happened accidentally due to plasticity of the thin brittle osteoporotic acetabulum floor which gave bouncing effect in every single hit during the cup placement. Paprosky classified our case as type III B acetabular defect [6]. He recommended the use of cemented cup on acetabular cage or reconstruction ring with bone allograft.

Since we did not expect for any complications, we do not have any availability of such cage implants. Therefore, in this emergency situation, we have to reconstruct the acetabulum floor with the unusual composite layers. The composite consisted respectively of remaining chondral bone shell, absorbable hemostatic gelatin sponge (Surgicell^®^) and sparse cancellous bone of the femoral head and porous collagen matrix (Osteovit^®^). On top of that, the uncemented titanium acetabulum cup (Reflexion^®^), which was failed when we attempted to anchore it in the soft acetabulum floor, was fixed with two screws, acting as reconstruction cage to cover the perforated acetabulum floor. We used Surgicell^®^ and Osteovit^®^ because they are always available in our operating theatre.

The aim of using the chondral bone shell of the femoral head together with Surgicell^®^ and Osteovit^®^ as the compound barrier wall between pelvic minor structure and the prosthesis was to create growing viable stable bony base as a seat of the acetabulum prosthesis. The study in vitro of the use of chondral shell shown that less than 10% loss of initial graft mass when using the whole femoral head without removing the articular cartilage, compared with the loss of 25% of graft mass when obtaining corticocancellous graft after removing cartilage [7]. The most common source for bone grafting material is fresh frozen femoral heads from bone bank, containing cortical bone of the neck, cancellous bone and remnants of articular cartilage [8]. However, according to other studies, fibrous ingrowth may also provide mechanical stability [9,10].

It has been shown that the acetabular allografts unite at an average of 11 months (range, 6–16 months) [11]. Paprosky et al. [12] reported a success rate of 82% in cases where a cage was used along with total acetabular allograft as compared with 38% in cases without the use of cage.

After we reconstructed the acetabulum floor with the composite layers, on top of that gentamicin contained bone cement was poured to fix the composite together with the cemented polyethylene cup (Reflexion All-poly^®^). After finishing the cemented acetabular component, we placed the cemented Synergy^®^ femoral prosthesis without any difficulty.

Postoperatively, the patient was advised to stay in bed for 6 weeks with passive exercise.

**Fig. 5:**
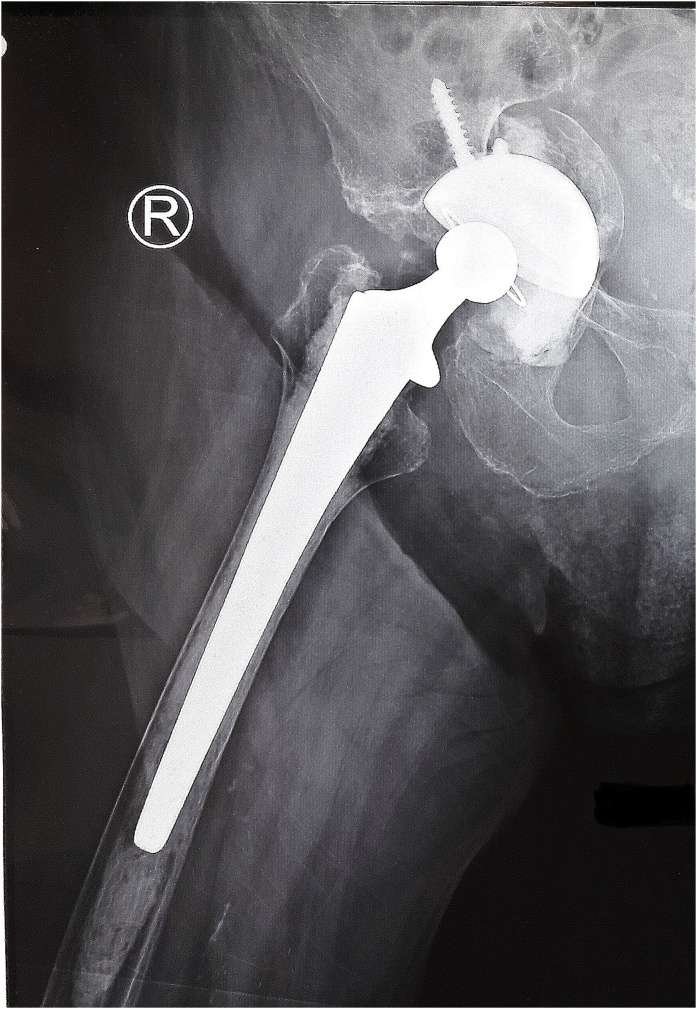
Fifteen months post-operative showed the stability of acetabulum composite graft (Source: internal documentation).

Active physiotherapy with partial weight-bearing using walker started thereafter. Patient was pain free and full ambulatory with walker at 4 months. X-ray showed the bone graft had united with the host bone and there is no evidence of any loosening, osteolysis, or resorption around the bone graft. After 15 months follow up (Fig. 5), Harris Hip Score showed 71.3% compared with 20.2% before surgery. There is no any previous use of similar technique we may find in the literature and to the best of our knowledge this technique also has not been mentioned for use in acetabular perforation in the literature. But this revision technique is appealing as it theoretically addresses the three main goals of acetabular reconstruction by paprosky : to convert uncontained defects to contained defects by using metal acetabulum cup that used as reconstruction cage, bone graft providing stable and viable contruct and restoring the center of rotation as close as possible.

## 4 Conclusion

In unexpected acetabulum perforation, the remaining chondral shell of femoral head together with uncemented cup as a cage support and bone cement can be used to reconstruct the acetabulum floor. More stability can be achieved using reconstruction cage with allograft and or metal augments or using a cemented total hip arthroplasty.

## Conflicts of interest

The authors have no conflict of interest to declare.

## Sources of funding

There is no specific grant from funding agencies in the public, commercial, or not-for-profit sectors.

## Ethical approval

Approval to publish case report is waived by the institution.

## Consent

Written informed consent was obtained from the patient for publication of this case report and accompanying images. A copy of the written consent is available for review by the Editor-in-Chief of this journal on request.

## Author’s contribution

Franky Hartono, Orthopaedic surgeon: the manuscript design and writing, the literature review and data collection.

Daniel Petrus Marpaung, Orthopaedic surgeon: the literature review.

Karina Besinga, Orthopaedic surgeon: the literature review.

Andrew Budiartha Budisantoso: the manuscript design and writing and data collection.

Tessi Ananditya: the manuscript writing.

## Registration of research studies

This is a Case Report.

## Guarantor

Franky Hartono.

## Provenance and peer review

Not commissioned, externally peer-reviewed.
